# Mechanism of extracellular space changes in cryptococcal brain granuloma revealed by MRI tracer

**DOI:** 10.3389/fnins.2022.1034091

**Published:** 2022-12-20

**Authors:** Nuerbiyemu Abulikemu, Xin Gao, Wei Wang, Qingyuan He, Gang Wang, Tao Jiang, Xiaodong Wang, Yumeng Cheng, Min Chen, Yanran Li, Lulu Liu, Jingjing Zhao, Jin Li, Chunhui Jiang, Yunling Wang, Hongbin Han, Jian Wang

**Affiliations:** ^1^Imaging Center, First Affiliated Hospital of Xinjiang Medical University, Ürümqi, China; ^2^Beijing Key Laboratory of Magnetic Resonance Imaging Devices and Technology, Peking University Third Hospital, Beijing, China; ^3^Institute of Medical Technology, Peking University Health Science Center, Beijing, China; ^4^Shanghai Universal Medical Imaging Diagnostic Center, Shanghai University, Shanghai, China; ^5^Department of Rehabilitation Radiology, Beijing Rehabilitation Hospital, Capital Medical University, Beijing, China; ^6^Department of Radiology, Peking University Third Hospital, Beijing, China; ^7^Imaging Center, Xi’an Gem Flower Changqing Hospital, Xi’an, China; ^8^The Animal Experimental Center, Xinjiang Medical University, Ürümqi, China; ^9^Department of Dermatology, First Affiliated Hospital of Xinjiang Medical University, Ürümqi, China; ^10^Department of Dermatology, Shanghai Key Laboratory of Molecular Medical Mycology, Changzheng Hospital, Second Military Medical University, Shanghai, China

**Keywords:** brain, cryptococcal granuloma, extracellular space, MRI tracer, mechanism

## Abstract

**Purpose:**

This study aimed to investigate the changes in extracellular space (ECS) in cryptococcal brain granuloma and its pathological mechanism.

**Materials and methods:**

The animal model of cryptococcal brain granuloma was established by injecting 1 × 10^6^ CFU/ml of *Cryptococcus neoformans* type A suspension into the caudate nucleus of Sprague–Dawley rats with stereotactic technology. The infection in the brain was observed by conventional MRI scanning on days 14, 21, and 28 of modeling. The tracer-based MRI with a gadolinium-diethylenetriamine pentaacetic acid (Gd-DTPA) as a magnetic tracer was performed on the rats with cryptococcal granuloma and the rats in the control group. The parameters of ECS in each area of cryptococcal brain granuloma were measured. The parameters of ECS in the two groups were compared by independent sample *t*-test, and the changes in ECS and its mechanism were analyzed.

**Results:**

Up to 28 days of modeling, the success rate of establishing the brain cryptococcal granuloma model with 1 × 10^6^ CFU/ml *Cryptococcus neoformans* suspension was 60%. In the internal area of cryptococcal granuloma, the effective diffusion coefficient D* was significantly higher than that of the control group (*t* = 2.76, *P* < 0.05), and the same trend showed in the volume ratio α (*t* = 3.71, *P* < 0.05), the clearance rate constant *k* (*t* = 3.137, *P* < 0.05), and the tracer half-life T_1/2_ (*t* = 3.837, *P* < 0.05). The tortuosity λ decreased compared with the control group (*t* = −2.70, *P* < 0.05). At the edge of the cryptococcal granuloma, the D* and α decreased, while the λ increased compared with the control group (D*:*t* = −6.05, *P* < 0.05; α: *t* = −4.988, *P* < 0.05; λ: *t* = 6.222, *P* < 0.05).

**Conclusion:**

The internal area of the lesion demonstrated a quicker, broader, and more extended distribution of the tracer, while the edge of the lesion exhibited a slower and narrower distribution. MRI tracer method can monitor morphological and functional changes of ECS in pathological conditions and provide a theoretical basis for the treatment *via* ECS.

## Introduction

Cryptococcal infection of the central nervous system (CNS) often occurs in immunocompromised patients, especially those with acquired immune deficiency syndrome (AIDS), and it can also be seen in immunocompetent individuals (Tan et al., 2016). CNS infections most commonly manifest as cryptococcal meningitis, but up to 14–25% of cases, especially those with a CD^+4^ cell count of < 50/mm^3^, may have brain parenchymal granulomatous lesions and worsen the prognosis of patients (Dzendrowskyj et al., 2005; Bowen et al., 2016; Paiva et al., 2018). Granuloma lesions of ≥ 3 cm in diameter need surgical resection, and lesions of < 3 cm in diameter are mainly treated with amphotericin B, fluorocytosine for induction therapy, and fluconazole, fluorocytosine for consolidation therapy (Perfect et al., 2010). However, due to the low diffuse rate of the drug across the blood–brain barrier (BBB), the amount of drug entering cerebrospinal fluid from peripheral blood is only 2–4% of the plasma concentration (Takemoto et al., 2006), and increased drug dose may lead to dose-dependent nephrotoxicity and systemic side effects, resulting in poor treatment efficacy in patients with cryptococcal granuloma.

Studies have shown that administration through the extracellular space (ECS) can avoid the BBB so that the drug can work directly on brain cells and achieve the best therapeutic effect at a dose far lower than that required in intravenous administration (Xu et al., 2011a). ECS is an ultra-fine lacunar structure between adjacent brain cells or cells and blood vessels, accounting for 15–20% of the volume of the living brain. It plays a role as a material transport channel in the process of drugs entering brain tissue through blood vessels and working on brain cells. Compared with the cerebrovascular system accounting for 3–5% of the volume of the living brain, ECS is more widely distributed and closely related to brain cells. It is closely involved in intercellular information transmission and cell migration and maintains the stability of the local brain microenvironment (Xu et al., 2011a; Lei et al., 2017; Soria et al., 2020). Therefore, monitoring the structural changes of the ECS under disease conditions is not only beneficial for a more precise understanding of mechanisms but also provides theoretical guidance for drug delivery through the ECS. The tracer-based MRI technique, which uses the T1 positive tracer as gadolinium-diethylene triaminepentacetic acid (Gd-DTPA) as the tracer probe, can reveal the diffusion law and pathway of the tracer *in vivo*. As the linear relationship between the concentration of Gd-DTPA and the signal intensity on the high-resolution T1-weighted 3-dimensional magnetization-prepared rapid-acquisition with gradient echo (T_1_WI 3D MP-RAGE) sequence has been confirmed (Xu et al., 2011b), the tracer-based MRI technique can quantitatively measure the diffusion parameters as well. It has been applied to detect ECS in the brain of glioma (Li et al., 2013), Parkinson’s disease (Ren et al., 2016; Fang et al., 2017), and Alzheimer’s disease (Liu et al., 2018; Yue et al., 2019) in rat models. However, study reports on ECS exploration of infectious brain granuloma are still missing. In this study, we use the tracer-based MRI technique to visually detect and quantitatively evaluate the diffusion process of tracer with interstitial fluid (ISF) in the brain of cryptococcal granuloma rats and analyze the alterations in ECS structure and ISF drainage in the cryptococcal granuloma, to provide a theoretical basis for the treatment of cryptococcal granuloma through the ECS.

## Materials and methods

### Animals

All experiments were performed on mature (8 weeks old, 200–250 g) female Sprague–Dawley rats (*n* = 40) and followed the guidelines for experimental animals. All experimental protocols were approved by the Experimental Animal Ethic Committee of Xinjiang Medical University (No. IACUC-20210115-33). Rats were randomly divided into a cryptococcal infected model group (*n* = 30) and a control group (*n* = 10).

### Fungal strain

The standard strain of serum type A *Cryptococcus neoformans* was donated by the Dermatology Department of the First Affiliated Hospital of Xinjiang Medical University. The strain was transferred to a YPD medium under sterile conditions, cultured at 37^°^ for 48–72 h, and diluted with saline into a fungal suspension with a concentration of 1 × 10^6^CFU/ml.

### Cryptococcal granuloma modeling

The procedure for cryptococcal granuloma modeling is as follows. First, the rats were anesthetized with Zoletil (10 mg/kg) combined with Xylazine Hydrochloride (10 mg/kg) by intramuscular injection, and the body temperature was maintained at 37 ± 0.5^°^C with a heating pad. The rats were fixed on the stereotactic apparatus (Lab standard Stereotaxic-Single, Stoelting, USA), an incision was made on the scalp along the sagittal suture, and the bregma was exposed. A small trephine hole in the skull bone was made according to the stereotactic coordinates of the caudate nucleus (bregma: + 1.0 mm, lateral: 3.5 mm, vertical: 5.0 mm) (Paxinos and Watson, 2007). A total of 10 μl of 1 × 10^6^ CFU/ml of fungal suspension was injected with a 10-μl microsyringe (Shanghai Gaoge, China) at a rate of 1 μl/min into the caudate nucleus of each rat in the cryptococcal granuloma model group using an automated drug administration system. After the injection, the needle was kept in place for 5 min and slowly withdrawn. The cranial hole was sealed with dental cement, and the incision was sutured. The rats were returned to the cage after they woke up and were raised under the original conditions.

### Monitoring of rat models

The rats injected with fungal suspension were scanned by a 3.0T MRI system (Skyra, Siemens Medical Solution, Germany) with an eight-channel wrist coil at 14, 21, and 28 days of modeling, and T_1_WI 3D MP-RAGE and T_2_WI sequence images were conducted to observe the infection. The scanning parameters were as follows: T_1_WI 3D MP-RAGE, repetition time (TR) = 1,500 ms, echo time (TE) = 3.7 ms, flip angle (FA) = 9°, inversion time (TI) = 900 ms, field of view (FOV) = 267 mm, voxel = 0.5 mm × 0.5 mm × 0.5 mm; T_2_WI, TR = 3620 ms, TE = 92 ms, FA = 120°, and FOV = 80 mm. According to the T_2_WI imaging findings, cryptococcal granuloma formation was suspected in rats with strip-shaped, flaky-shaped, round, or nodular-like lesions that displayed hypointensity on T_1_WI and hyperintensity on T_2_WI. Since the conventional imaging manifestation of postoperative reaction is similar to that of suspected granuloma, the modeling results should be subject to the scanning results on the 28th day.

### Tracer-based MRI scanning

Rats with cryptococcal granuloma and those in the control group were scanned with the tracer-based MRI technique. Anesthesia was performed as described earlier. Pre-scan was performed with T1WI 3D MP-RAGE sequences (the scanning parameters were the same as those of rat model monitoring) before tracer injection and recorded as T0. After pre-scan, a 2 μl volume of 10 mmol/L Gd-DTPA solution (Consun Pharmaceutical Group, Guangzhou, China, 20 ml/9.38 g) was microinjected into the caudate nucleus with a 2-μl microsyringe (Shanghai Gaoge, China) at a rate of 0.2 μl/min using an automated drug administration system. The injection was followed by a 5-min waiting period to avoid dorsal reflux along the needle track. The rats were then placed in the scanner in a prone position for a post-injection scan. Repeated MRI T1WI 3D MP-RAGE sequences scanning was performed at 15, 30, 45, 60, 80, 100, 120, 150, 180, and 240 min post-injection (Han et al., 2014).

### ECS diffusion parameters measurement

The ISF flow was observed according to a previously reported method (Wang et al., 2019). The ISF diffusion and ECS structural parameters were analyzed using the Nano Imaging Detect Analyze System, Version 2.1 (MRI lab, Beijing, China) (Wang et al., 2019; Gao et al., 2021). Once a region of interest (ROI) was selected, the MRI signal intensity was the average pixel intensity in the ROI. Post-injection images were compared with the baseline images after grayscale calibration, mutual information-based image registration, and histogram equalization. After a series of image pre-processing (including rigid transformation, similarity measurement, high-order interpolation, and adaptive stochastic gradient descent optimization), all post-injection images were subtracted from the pre-scanned images. Post-processing MR images in the horizontal, sagittal, and coronal planes were generated by the software, and the signal intensity within the ROI of the processed images was measured and denoted as the increment in the signal intensity (Wang et al., 2019). Since the linear relationship between Gd-DTPA concentration and signal intensity in the T1WI 3D MP-RAGE sequence has been confirmed (Xu et al., 2011b), the concentration of GD-DTPA in ECS can be calculated in real time according to the change in signal intensity to obtain the diffusion coefficient (D*), volume fraction (α), tortuosity (λ), clearance rate constant (*k*), and half-life (T_1/2_). The D* is the effective diffusion coefficient of a given molecule in the brain ECS. It was calculated according to the concentration of the tracer diffused to the r region at time t, where r is the diffusion radius. The volume fraction (α) was defined as the ECS volume fraction of the whole brain tissue. The tortuosity (λ) = (D/D*)^1/2^, where D is the diffusion coefficient of the same molecule in the free medium and the D of the tracer we used is 1.06 × 10^–3^ mm^2^/s (Li et al., 2013). The λ is the hindrance to diffusion caused by the ECS structure. The clearance rate constant (*k*) and half-life (T_1/2_) measure the loss of the molecule from ECS. A more detailed description of data processing and mathematical analysis can be found in prior articles (Xu et al., 2011b; Han et al., 2014; Wang et al., 2019). Images with tracer reflux, a bubble at the injection point, insufficient tracer dose, and other factors affecting parameter measurement shall be excluded.

### Pathological examination

The rats were sacrificed after tracer-based MRI scanning and taken out of the brain tissue on the operation platform of the biosafety cabinet, fixed with 10% formaldehyde for 48–72 h, dehydrated, and embedded in paraffin for the pathological section and hematoxylin-eosin staining. The histopathological changes in the lesion area were observed under the microscope.

### Statistical analysis

Statistical analyses were conducted using SPSS software, version 22.0 (IBM, USA). Data were expressed as the mean ± standard deviation. An independent sample t-test was used to compare the mean of the two groups. The *p*-value of < 0.05 was used as the threshold for statistical significance.

## Results

### Modeling result

On the 28th day of modeling, 18 rats (60%) were suspected of cryptococcal granuloma formation in the caudate nucleus area by 3D MP-RAGE T1WI and T2WI scanning, two rats (6.67%) died after surgical procedure, and the remaining 10 rats (33.33%) only had meningitis changes or no apparent abnormality. The granuloma formation rate of 1 × 10^6^CFU/ml serum type A *Cryptococcus neoformans* suspension was 60%.

### Analyses of ECS diffusion parameters

**FIGURE 1:**
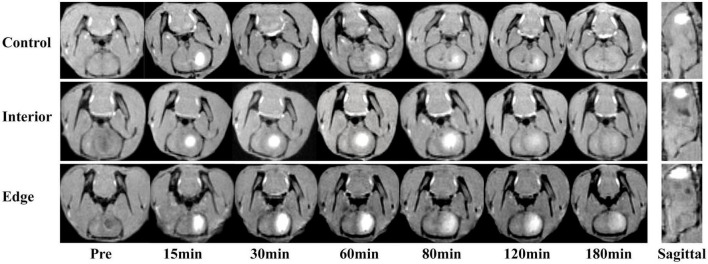
Coronal and sagittal images after Gd-DTPA injection. Images were selected according to a maximum range of Gd-DTPA. Sagittal images demonstrated the relationship between Gd-DTPA and the lesion.

**FIGURE 2:**
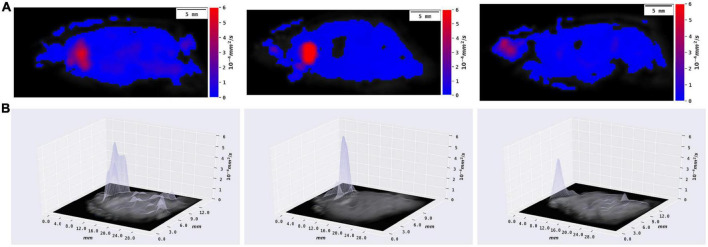
From left to right: control, granuloma interior, and granuloma edge. **(A)** Two-dimensional mapping indicated that the diffusion range increased in the granuloma interior group and decreased in the granuloma edge group. Scale bar = 5 mm. **(B)** Three-dimensional mapping showed that the diffusion level increased in the granuloma interior group and decreased in the granuloma edge group. Scale bar = 5 mm.

**FIGURE 3:**
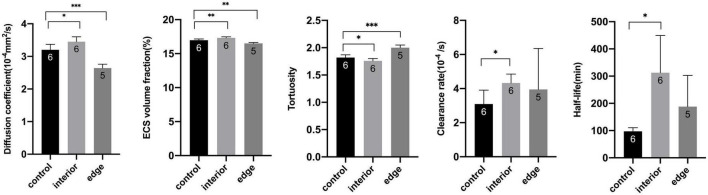
Statistical analysis of diffusion parameters between groups. *P < 0.05, ^**^P < 0.01, and ^***^P < 0.001.

After image quality screening, the images of 11 rats were qualified for ECS parameter measurement. Among them, the tracer in six rats was injected into the lesion interior area and five in the edge area. Because of the tremendous pathological difference between the interior and the edge of granuloma, they were analyzed separately. Figure 1 shows the tracer drainage of three groups over time, and the diffusion level is shown in Figure 2. The ECS parameters of the granuloma interior, granuloma edge, and control group, as well as the *t*-test results, are shown in Table 1 and Figure 3.

**TABLE 1:** ECS parameters and *t*-test results of granuloma interior, edge, and control group.

Parameters	Control group	Granuloma interior	Granuloma edge	*P* (interior vs. control)	*P* (edge vs. control)
D* (× 10^–4^ mm^2^/s)	3.20 ± 0.17	3.45 ± 0.15	2.64 ± 0.12	0.02*	0.000***
α%	16.98 ± 0.18	17.33 ± 0.15	16.51 ± 0.13	0.004**	0.001**
λ	1.82 ± 0.05	1.76 ± 0.04	2.01 ± 0.05	0.022*	0.000***
k (× 10^–4^/s)	3.09 ± 0.82	4.33 ± 0.52	3.95 ± 2.40	0.011*	0.478
T_1/2_ (min)	97.33 ± 13.39	312.66 ± 136.82	188.12 ± 114.67	0.012*	0.152

D*, diffusion coefficient; α, volume fraction; λ, tortuosity; k, clearance rate constant; T_1/2_, half-life.

*P < 0.05, **P < 0.01, and ***P < 0.001.

In the internal area of granuloma, the volume ratio α of the internal area was (17.33 ± 0.15)% and increased compared with the control group [(16.98 ± 0.18)%, *t* = 3.71, *P* < 0.05], indicating that the volume of the ECS in the internal area had increased. The tortuosity λ was 1.76 ± 0.04 and significantly lower than that of the control group (1.82 ± 0.05, *t* = −2.70, *P* < 0.05), which means the diffusion resistance in the internal area had decreased. The effective diffusion coefficient D* was (3.45 ± 0.15) × 10^–4^ mm^2^/s and significantly higher than that of the control group [(3.20 ± 0.17) × 10^–4^ mm^2^/s, *t* = 2.76, *P* < 0.05], indicating that the diffusion rate had increased in the internal area of the lesion, and the clearance rate constant *k* showed the same trend [(4.33 ± 0.52) × 10^–4^/s vs. (3.09 ± 0.82) × 10^–4^/s, *t* = 3.137, *P* < 0.05]. However, the tracer half-life T_1/2_ was prolonged ((312.66 ± 136.82) min vs. (97.33 ± 13.39) min, *t* = 3.837, *P* < 0.05).

In the edge area of granuloma, the volume ratio α was (16.51 ± 0.13)% and lower than that of the control group [(16.98 ± 0.18)%, *t* = −4.988, *P* < 0.05], indicating that the volume of the ECS in edge area had decreased. The tortuosity λ was significantly higher than that of the control group (2.01 ± 0.05 vs. 1.82 ± 0.05, *t* = 6.222, *P* < 0.05), which can be caused by decreased volume. The effective diffusion coefficient D* was significantly lower than that in the control group [(2.64 ± 0.12) × 10^–4^ mm^2^/s vs. (3.20 ± 0.17) × 10^–4^ mm^2^/s, *t* = −6.05, *P* < 0.05].

### Pathological findings

**FIGURE 4:**
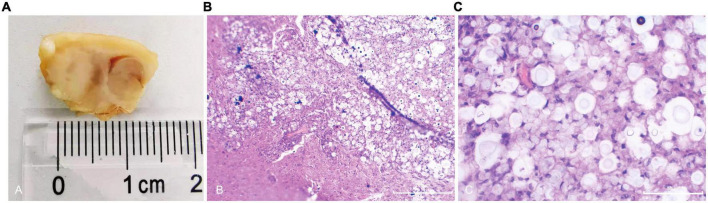
General and microscopic pathological observations of cryptococcal granuloma of the brain. In macroscopic view **(A)**, a mass necrotic focus can be seen in the caudate nucleus area with thick exudate on the surface; under 10 × 10-fold microscope **(B)** with HE staining, multiple cysts and inflammatory cell infiltration in brain parenchyma were seen; under the 40 × 10-fold microscope **(C)** with HE staining, a large number of circular cryptococci were scattered in the brain parenchyma, and some brain tissues were dissolved.

Brain biopsy of rats with cryptococcal granuloma showed thick yellow exudates, and necrotic foci can be seen in the caudate nucleus and part of the brain surface. Granuloma foci composed of lymphocytes and multinucleated giant cells can be seen under the microscope, with many cryptococci neoformans (Figure 4). Some rats’ ipsilateral meninges and ependyma were thickened to varying degrees, and *Cryptococcus neoformans* infiltrated some thickened areas.

## Discussion

Brain ECS is the microenvironment for the survival and metabolism of brain cells. It plays a major role in the occurrence and development of brain diseases and is also a channel that can bypass the BBB and treat brain diseases. To our knowledge, this is the first study to explore the structure of ECS and the mechanism of ISF drainage in infectious brain granuloma. The results may provide a theoretical basis and guidance for treating cryptococcal brain granuloma *via* ECS.

We found that the changes in ECS structure and diffusion parameters of the interior and edge area of the cryptococcal granuloma lesion were quite different. Inside the granuloma lesion, D*, α, and *k*-value, which, respectively, characterize the diffusion rate, volume fraction, and clearance rate of local ECS, increased than that of the control caudate nucleus. Moreover, tortuosity λ, which characterizes the diffusion resistance of ECS within the lesion, was lower than that in the control caudate nucleus. One possible reason for these results is related to the lysis and necrosis of the brain cells inside the granuloma lesion. After cryptococcus enters the brain through the BBB and causes an allergic reaction, proteolytic enzymes are released from dead inflammatory cells to dissolve and necrosed the brain tissue. Brain cell fragments and cryptococcus appear in the necrotic area, and the structure of normal brain cells disappears (Thomas and Sandritter, 1989; Charlier et al., 2009). Hence, when Gd-DTPA was injected into the interior area of the lesion, the diffusion resistance caused by brain cells, extracellular matrix, and ECS circuitous structure decreased significantly, causing the increase of the diffusion rate and expansion of the diffusion range. The necrotic area is surrounded by macrophages, lymphocytes, and other immune cells, accompanied by hyperemic small blood vessels and inflammatory cell infiltration, forming dense granulomas and restricting fungal dissemination (Thomas and Sandritter, 1989). Therefore, the rates of local spread and clearance within ECS lesions increased. Nevertheless, the half-life T_1/2_ was significantly prolonged due to the granulomatous wall limitation. Fang et al. (2017) used the tracer-based MRI method to explore the pathological changes of ECS in Parkinson’s disease rats and reached a similar conclusion: Due to the necrosis of dopamine neurons, the tortuosity of ECS at the substantia nigra of PD rats decreased, and the diffusion coefficient increased. However, accumulated neuronal metabolic waste produced by PD rats will hinder the drainage of ISF, resulting in the prolongation of half-life. At the edge of the granuloma lesion, D* and α decreased than that of the control caudate nucleus and λ increased. Two potential rationales can explain these observations. First, the increased cell density caused by gathered immune cells at the granuloma wall and the marginal area may be responsible for these findings. With the increase in cell density, the space between the adjacent cells narrowed, resulting in decreased ECS volume, increased ISF diffusion obstacles, and geometric diffusion path, eventually leading to increased tortuosity and reduced diffusion rate (Zuo et al., 2015). Second, cryptococcus tends to gather in ECS and continuously proliferate while secreting colloidal exudates, blocking the ECS pathway, and increasing ISF diffusion resistance.

Previous research (Fang et al., 2017) showed that the *k*-value has a negative correlation with the T_1/2_. Nevertheless, in this study, the *k*-value increased locally due to the increase in diffusion rate inside the lesion, while the T_1/2_ was also significantly prolonged due to the presence of a double-layer dense granuloma wall which restricts the diffusion of ISF. According to the results, we can preliminarily speculate that although the diffusion rate of cryptococcus inside the necrotic area of granuloma increases, due to the restricted role of the granuloma wall, the rate of cryptococcal infiltration into normal brain parenchyma is low.

Part of the lack of efficacy of intravenous administration against CNS diseases results from the fact that therapeutic agents with a molecular weight greater than 180 kDa cannot pass through the BBB (Jahangiri et al., 2017). Intraparenchymal delivery, which precisely delivers drugs to the target area *via* ECS, can overcome this difficulty. Many studies have verified its treatment effectiveness in animal and clinical studies of glioma (Laske et al., 1997; Voges et al., 2003; Weaver and Laske, 2003; Lidar et al., 2004; Bruce et al., 2011), Parkinson’s, (Gill et al., 2003; Marks et al., 2008, 2010) and Alzheimer’s diseases (Ksendzovsky et al., 2012). Thereby, we assumed that drug delivery *via* ECS could be a potential treatment for cryptococcal granuloma. When the drug is injected into the granuloma *via* ECS, the distribution speed of the drug in the granuloma is fast, the residence time is prolonged, and the cryptococcus can be effectively removed. The solute drainage speed at the edge of granuloma is slow, and the half-life has not been significantly prolonged. Therefore, when injecting drugs into the edge of a granuloma, the drug dose can be appropriately increased according to individual conditions to prolong the drug distribution time.

The main approaches for detecting the ECS *in vivo* include real-time ion introduction, integrated optical imaging, and MRI tracer imaging (Lei et al., 2017). Although the first two methods can measure the morphological parameters of ECS in the local brain tissue (60–100 μm) (Sykova and Nicholson, 2008), they cannot be used to detect and monitor the ECS in the deep center of the brain due to their limitation of detecting depth and low image resolution. With the advantages of high soft tissue resolution, real-time monitoring, and global imaging, MRI is unique in detecting and imaging the brain ECS at the whole brain scale and is used to estimate the diffusion and tortuosity values of ECS. However, ECS research is a complex project that involves MRI protocol and probe, signal detection and processing, and mathematical models, and different approaches have different strengths and limitations. Marty et al. (2013) injected a T_1_ relaxing probe and set up a 2D diffusion model to estimate the effective diffusion coefficient D* which is determined from the rate of increase of the spatial width of the Gaussian distribution of the probe in the tissue. The result showed that the measured D* value for gadoteric acid and the tortuosity factor was inconsistent with the results obtained from model prediction and optical method. We assumed this was because a 2D diffusion model cannot simulate the anisotropic diffusion of ISF and a low temporal sample could fail to achieve a satisfactory dynamic range and observe the complete diffusion time course. These two problems were solved in Hagberg et al. (2014) research, which yielded whole brain coverage with a high spatial and temporal sampling and obtained D* values that were consistent with optical measurement by the voxel-wise fitting of the solution to the diffusion equation. However, the method proposed in Hagberg’s research was not able to assess volume ratio α, since the *in vivo* relaxivity of the compartments cannot be confirmed and the signals from the extra- and intracellular compartments cannot be separated. In this study, we used Gd-DTPA as a probe and demonstrated a 3D visualization of the dynamic drainage flow of brain ISF in a global view, as well as anisotropic diffusion properties in brain ISF (Wang et al., 2019). As an extracellular contrast agent, Gd-DTPA induces signal enhancement by alerting the proton relaxation of ISF. The signal increment and its time course can be used to obtain the tracer concentration at any point and time (Han et al., 2014). The principle of D* value measurement in tracer-based MRI is similar to that of Hagberg’s, which is calculating the voxel by voxel-based on a modified diffusion model. Moreover, the obtained D* value was in agreement with the RTI-TMA^+^ method. In addition, the volume ratio α can be obtained in our method since the linear relationship between the signal increment and Gd-DTPA concentration on 3D MP-RAGE sequences has been confirmed and the relaxivity of Gd-DTPA can be assessed according to the linear curve. In addition, as the Gd-DTPA is retained mainly in the ECS, an enhanced extracellular region can be separated from the unenhanced cellular region (Xu et al., 2011b). However, compared with Hagberg’s method, the temporal sample of our method was relatively low and it may reduce the accuracy of the parameters. This limitation could be improved in future research by multiple MR scanning till the tracer faded completely.

Although the rate of granuloma formation was 60% (18/30), which was quite high, only the images of 11 rats were qualified for ECS parameters measurement. The images of the other seven rats were excluded for the following limitations. First, MRI tracer scanning included invasive operations and required multiple additional anesthesia for a longer duration of scanning, which caused a cerebral hemorrhage in two rats and respiratory inhibition in one rat. The scanned images were insufficient for the measurement of ECS parameters due to scanning interruption. Second, images of four rats were excluded due to the occurrence of tracer reflux, a bubble in the injection point, and artifacts in several scan points since these factors could decrease the tracer dosage and cause measurement error. Nevertheless, the enrolled images of these 11 rats are adequate to represent the characteristics of the disease as they were obtained through multi-level strict quality control.

## Conclusion

The extracellular space structure of the granuloma interior area disappeared, and the tracer diffused more unobstructedly, while the granuloma wall restricted the diffusion. There are many disadvantages in the traditional delivery of drugs to treat brain cryptococcal granuloma, while surgical treatment is risky. Therefore, drug delivery *via* ECS to treat brain cryptococcal granuloma has great clinical significance, and the findings of this study have important implications for the treatment of cryptococcal granuloma *via* ECS as a new approach to drug delivery.

## Data availability statement

The raw data supporting the conclusions of this article will be made available by the authors, without undue reservation.

## Ethics statement

This animal study was reviewed and approved by the Experimental Animal Ethic Committee of Xinjiang Medical University (No. IACUC-20210115-33).

## Author contributions

NA: literature review, research design, experiment implementation, data acquisition, data analysis, manuscript drafting, figures and table arrangement, and manuscript submission. XG: research design, consumables acquisition, experimental guidance, data analysis, and writing—review. WW: research design, experimental guidance, data analysis, writing—review, and revision. QH: experimental guidance, data acquisition, data analysis, and writing—review. GW: research design, experiment implementation, and data acquisition and analysis. TJ: research design, consumables acquisition, and experimental guidance. XW: consumables acquisition and experimental guidance. YC: experimental guidance and data acquisition and analysis. MC: consumables acquisition and experimental guidance. YL, LL, and JZ: experiment implementation. JL: experiment implementation and data sorting. CJ: experimental guidance, data acquisition, and writing—review. YW: research design, experiment implementation, and writing—review. HH: research design and guidance, technical provision, data analysis, writing—review, and quality control. JW: conceptualization, resources, research design, supervision, writing—review, quality control, final approval of this manuscript, and project administration. All authors contributed to the article and approved the submitted version.
